# Age-Related Cutoffs of Plasma Aldosterone/Renin Concentration for Primary Aldosteronism Screening

**DOI:** 10.1155/2018/8647026

**Published:** 2018-07-16

**Authors:** Linqiang Ma, Ying Song, Mei Mei, Wenwen He, Jinbo Hu, Qingfeng Cheng, Ziwei Tang, Ting Luo, Yue Wang, Qianna Zhen, Zhihong Wang, Hua Qing, Yihong He, Qifu Li, Shumin Yang, the Chongqing Primary Aldosteronism Study (CONPASS) Group

**Affiliations:** ^1^Department of Endocrinology, The First Affiliated Hospital of Chongqing Medical University, Chongqing, China; ^2^Department of Radiology, The First Affiliated Hospital of Chongqing Medical University, Chongqing, China

## Abstract

**Aim:**

This retrospective study is aimed at investigating whether aldosterone-renin ratio (ARR) cutoffs calculated by the plasma aldosterone concentration (PAC)/plasma renin concentration (PRC) should be set differently in patients of different ages.

**Methods:**

521 hypertensive patients were screened for primary aldosteronism (PA) by the PAC/PRC. 174 patients diagnosed with PA and 311 patients with essential hypertension (EH) were included in the final analysis. Subjects were subdivided into four age groups: <40, 40–49, 50–59, and ≥60 years old.

**Results:**

The accuracy of the ARR varied greatly among the different age groups. An ARR of 3.7 (ng/dl)/(*μ*IU/ml) had a sensitivity of 100% and a specificity of 80% in patients ≥ 60 years old. With this cutoff, the sensitivities in patients < 40, 40–49, and 50–59 years old were 74%, 82%, and 87%, respectively, and the specificities were 94%, 95%, and 94%, respectively. To achieve a sensitivity higher than 90%, the ARR cutoff needed to be lowered to 2.0 (ng/dl)/(*μ*IU/ml) for patients 40–49 and 50–59 years old, resulting in sensitivities of 90% and 95%, respectively, and specificities of 80% and 84%, respectively. To achieve a sensitivity higher than 90%, the ARR cutoff needed to be lowered to 1.0 (ng/dl)/(*μ*IU/ml) for patients < 40 years old, resulting in a sensitivity of 90% and a specificity of 82%.

**Conclusions:**

An ARR of 3.7 (ng/dl)/(*μ*IU/ml) is optimal for patients ≥ 60 years; for patients 40–59 years, the optimal ARR cutoff is 2.0; for those younger than 40 years, an ARR of 1.0 may be more reasonable.

## 1. Introduction

Primary aldosteronism (PA) is caused by adrenocortical lesions and is characterized by autonomous secretion of aldosterone. The aldosterone-renin ratio (ARR) is advocated as the most reliable tool for PA screening in high-risk groups of hypertensive patients [1]. However, appropriate ARR cutoffs should be selected cautiously and validated clinically, as the ARR varies substantially depending on the analytical methods used [2]. Conventionally, plasma renin activity (PRA) is measured by radioimmunoassay to calculate the ARR; however, the plasma renin concentration (PRC), which is measured by an automated chemiluminescent immunoassay, has become increasingly popular because this procedure is less laborious and time consuming [3, 4]. Previous studies have revealed that PRA and PRC were well correlated and that the automated renin chemiluminescent assay was a reliable alternative to the radioimmunometric method [5–8]. Our previous meta-analysis that analyzed the accuracy of PAC/PRC as a screening test in patients with PA revealed that the overall sensitivity, specificity, and area under the curve of PAC/PRC were 0.89, 0.96, and 0.985, respectively, demonstrating the efficacy of PAC/PRC as a screening test for PA [9]. Currently, there are no unanimous cutoff values for the ARR for PA screening, and the PRC-based ARR cutoffs mentioned in previous studies varied between 1.0–5.7 (ng/dl)/(*μ*IU/ml) [3–8, 10–14].

Globally, the population is aging and the World Health Organization predicts that by 2050, the population aged 60 years or older will double. PA is more common in young or middle-aged hypertensive patients; however, as the population ages, PA screening may become more popular in elderly patients with hypertension. It is generally accepted that both the plasma renin and aldosterone levels tend to fall with advancing age and plasma renin decreases more than aldosterone; consequently, the ARR increases in the elderly [1]. Thus, using the same ARR cutoff in patients of different ages for PA screening might cause false positive results and render unnecessary confirmatory testing, such as saline infusion testing, which carries a potential risk for acute volume overload in elderly patients. Several studies have focused on the effects of age on PA screening but drew inconsistent conclusions. Yin et al. [15] reported that the cutoffs for the ARR for PA screening were not affected by age, while two other studies [16, 17] suggested that the ARR criteria might need to be higher with advancing age. However, the ARR in these studies was based on the PRA and few studies have evaluated the influence of aging on the ARR cutoffs calculated with the PRC.

In this retrospective study, ARR cutoffs based on PRC were evaluated in 485 hypertensive patients and whether or not ARR cutoffs should be set differently in patients of different ages for PA screening was investigated.

## 2. Methods

### 2.1. Participants

This study retrospectively analyzed data from a previously published prospective study that was conducted in the Department of Endocrinology at the First Affiliated Hospital of Chongqing Medical University from November 2013 to September 2016 [18]. The protocol was approved by the ethics committee of the First Affiliated Hospital of Chongqing Medical University. Written informed consent was obtained from each participant. This prospective study is aimed at evaluating the diagnostic accuracy of the saline infusion test and the captopril challenge test, using the fludrocortisone suppression test as the reference standard, in patients with a high risk for PA according to the following criteria: Joint National Commission stage 2 (blood pressure, BP > 160–179/100–109 mm Hg), stage 3 (BP > 180/110 mm Hg), or drug-resistant hypertension; hypertension and spontaneous or diuretic-induced hypokalemia; hypertension with adrenal incidentaloma; hypertension and a family history of early-onset hypertension or cerebrovascular accident at a young age (<40 years); or a hypertensive first-degree relative of PA.

The exclusion criteria were as follows: patients diagnosed with secondary hypertension other than PA and patients who screened positive but failed to complete any confirmatory testing and did not have a conclusive diagnosis. However, patients with spontaneous hypokalemia, undetectable renin concentrations, and a PAC > 20 ng/dl were included as PA patients, even though no further confirmatory testing was performed.

### 2.2. Screening

For screening tests, treatment with diuretics was withdrawn for at least four weeks, and *β*-blockers, angiotensin-converting enzyme inhibitors, and angiotensin-1 receptor blockers were stopped for at least two weeks. Only nondihydropyridine calcium channel blockers, terazosin, and doxazosin were allowed for uncontrolled hypertension. Samples for the PRC and PAC were collected in the morning after patients were out of bed for at least 2 h. The screening test was considered positive when the ARR was ≥3.7 (ng/dl)/(*μ*IU/ml).

### 2.3. Diagnosis

Patients who tested positive or who tested negative but in whom PA was strongly suspected (e.g., recurrent hypokalemia and resistant hypertension) underwent the confirmatory tests (saline infusion test, captopril challenge test, or fludrocortisone suppression test). For the remaining patients who screened negative, one of every three consecutive patients underwent the confirmatory tests, which were performed on three separate days.

The saline infusion test was conducted as follows: patients stayed in the recumbent position for at least 1 h before and during the infusion of 2 liters of 0.9% saline over 4 h, starting at 8 a.m. Blood samples to measure the PRC, PAC, cortisol, and plasma potassium were drawn at time zero and after 4 h; during the test, patients fasted, and their blood pressure and heart rate were strictly monitored.

The captopril challenge test was conducted as follows: patients received 50 mg of oral captopril at 8-9 a.m. after sitting or standing for at least 1 h. Blood samples were drawn for measurement of the PRC and PAC at time zero and at 2 h after the challenge, with patients remaining seated during this period.

The fludrocortisone suppression test was conducted as follows: patients received 0.1 mg of oral fludrocortisone every 6 h for 4 d, together with slow-release potassium chloride (KCl) supplements to maintain plasma K^+^ levels close to 4.0 mmol/liter, sodium chloride (NaCl) supplements (30 mmol thrice daily with meals), and sufficient dietary salt to maintain a urinary sodium excretion rate of at least 3 mmol/kg. On day four, the PAC and PRC were measured at 10 a.m. with the patient in a seated posture and the plasma cortisol was measured at 7 a.m. and 10 a.m.

The diagnosis of PA was established according to the fludrocortisone suppression test criteria [19, 20]: when the PAC was not suppressed to less than 8 ng/dl (220 pmol/l) on day four, the diagnosis of PA was considered. In addition, if adrenal venous sampling (AVS) was performed despite a negative FST and demonstrated lateralization that led to unilateral adrenalectomy, these patients were included in the PA group.

For patients who tested positive during screening but did not undergo the fludrocortisone suppression test, if the postinfusion PAC was over 8 ng/dl during the saline infusion test or the PAC suppression ratio was higher than 11 ng/dl during the captopril challenge test, PA was considered [18]. For subtyping, the diagnosis of aldosterone-producing adenoma required fulfillment of all of the following criteria: [1] unilateral excessive aldosterone secretion confirmed by AVS and/or evidence of a unilateral adrenocortical nodule on CT scan, [2] pathologically confirmed adenoma after surgery, and [3] complete biochemical success during the postadrenalectomy follow-up [21].

**Figure 1:**
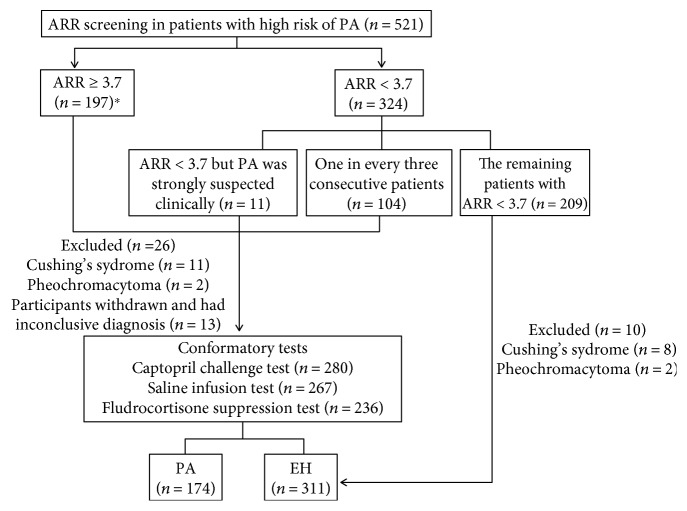
Flow of participants. ^∗^Six patients with spontaneous hypokalemia, undetectable renin, and PAC > 20 ng/dl, though no further confirmatory testing was performed, were included as PA.

In patients with confirmed PA onset at younger age (earlier than 20 years) and in those who had a family history of PA or of strokes at a young age, chimeric CYP11B1/CYP11B2 genetic testing for glucocorticoid remediable aldosteronism was performed with a long polymerase chain reaction technique [22]. A dexamethasone suppression test and catecholamine assays were performed to rule out hypercortisolism and pheochromocytoma. The flow of participants was shown in Figure 1.

### 2.4. Biochemical Measurements

The PRC and PAC were measured with an automated chemiluminescence immunoassay (LIAISON; DiaSorin, Italy). The analytical sensitivity (defined as the minimum detectable dose that could be distinguished from zero) for the PRC was 0.53 *μ*IU/ml, and the functional sensitivity (defined as the concentration at which the between-assay coefficient of variation exceeded 20%) for the PRC was 1.6 *μ*IU/ml. The intra-assay coefficient of variations (repeatability) and interassay coefficient of variations (reproducibility) for the PRC ranged from 1.2% to 3.7% and 2.9% to 12.8%, respectively. The PAC assay is capable of measuring a range from 2.2 ng/dl (analytical sensitivity) to 100 ng/dl, with a functional sensitivity of 3 ng/dl. The intra-assay coefficient of variations for the PAC ranged from 2.4% to 4.8%. The interassay coefficient of variations ranged from 4.4% to 6.7%. Quality control was performed every day in the laboratory.

### 2.5. Statistical Analysis

SPSS 21 was used for statistical analysis. The results below the detection level were set to each assay's respective analytical sensitivity value for comparative purposes. The distribution of the data was analyzed with the Kolmogorov-Smirnov test. Normally distributed variables were expressed as the mean ± standard deviation (SD); variables with a skewed distribution were expressed as the median (quartile range); categorical variables were described as percentages. Variables with a skewed distribution were analyzed after a natural logarithm transformation. Associations between age and the PRA, PAC, and ARR were calculated with Spearman correlations. One-way ANOVA was used to analyze the trends of the PRA, PAC, and ARR with changes in age. Categorical variables were analyzed with *χ*2 test, and quantitative variables were analyzed with Student's *t*-test. To assess the diagnostic accuracy of the screening tests, parameters including the sensitivity and specificity were calculated. *P* values < 0.05 (two-tailed) were considered statistically significant.

## 3. Results

### 3.1. Clinical Characteristics of the Subjects

Five hundred and twenty-one hypertensive patients were screened for PA. Thirty-six patients were excluded from the analysis: 23 patients diagnosed with other causes of secondary hypertension and 13 patients with inconclusive diagnoses. Six patients with spontaneous hypokalemia, undetectable renin concentrations, and PAC > 20 ng/dl were included as PA, even though no further confirmatory testing was performed. Overall, 174 patients diagnosed with PA and 311 patients diagnosed with essential hypertension (EH) were included in the analysis (Figure 1).

The clinical characteristics of the subjects are shown in Table 1. There were no significant differences between the EH and PA groups in terms of gender or age. Patients with PA had higher levels of SBP, DBP, PAC, ARR, serum sodium, and 24 h urine potassium, whereas those with PA had lower serum potassium and PRC levels.

**Figure 2:**
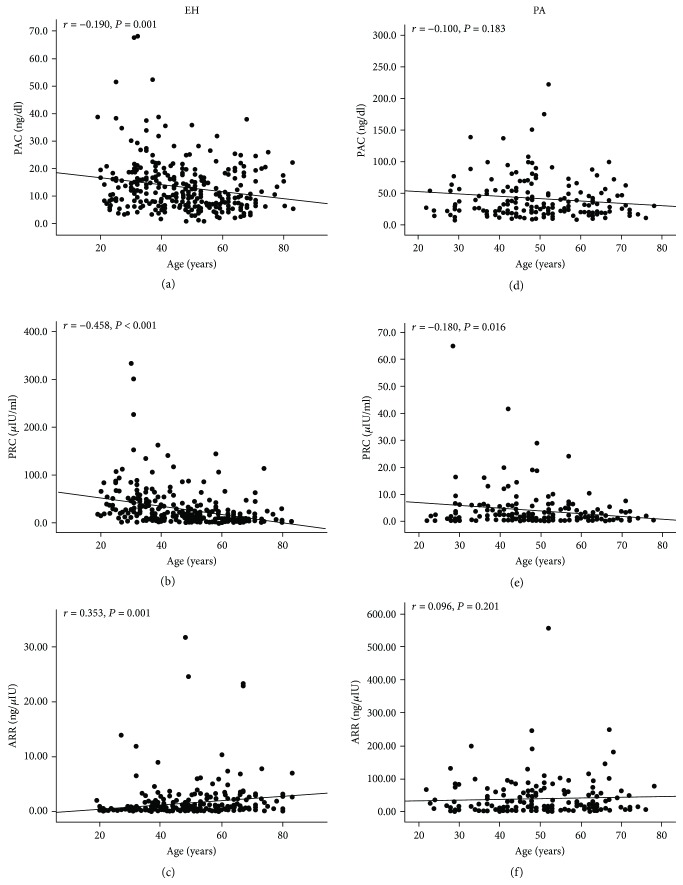
Scatter plots showing the correlation of PRC, PAC, and ARR levels with age in patients with EH and PA. The PAC (a) and PRC (b) were negatively correlated with age whereas ARR (c) was positively correlated with age in patients with EH. In the PA group, PRC was negatively correlated with age but neither PAC nor ARR was significantly associated with age (d–f).

The PRC and PAC were negatively associated with age (*r* = −0.46, *P* < 0.001 and *r* = −0.19, *P* < 0.001), whereas the ARR was positively associated with age (*r* = 0.35, *P* < 0.001) in the EH group (Figures 2(a)–2(c)). In the PA group, the PRC was negatively associated with age (*r* = −0.18, *P* = 0.016), while neither the PAC (*r* = −0.10, *P* = 0.18) nor the ARR (*r* = 0.10, *P* = 0.20) were significantly associated with age (Figures 2(d)–2(f)).

**Figure 3:**
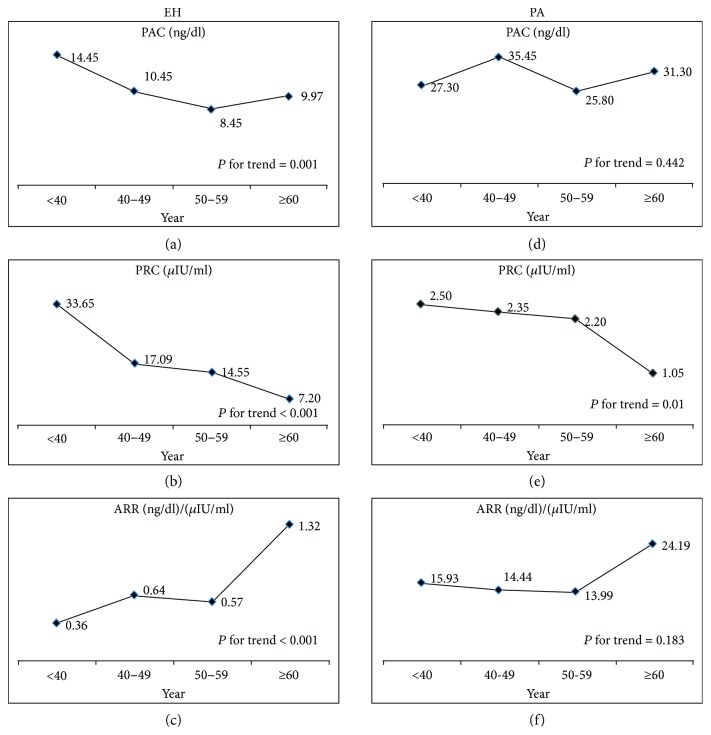
Line chart of PAC, PRC, and ARR in different age groups in patients with EH and PA. In the EH group, PRA lowered more than PAC and led to higher ARR with increasing age. In the PA group, PRA significantly declined with increasing age, but the trends for PAC and ARR were not significant. ARR: aldosterone to renin ratio; PA: primary aldosteronism; PAC: plasma aldosterone concentration; PRC: plasma renin concentration.

To analyze the tendency of changes in the PAC, PRA, and ARR in different age groups, subjects were subdivided into four groups as follows: <40, 40–49, 50–59, and ≥60 years old. The clinical characteristics of the subjects in the different age subgroups are shown in Table 2. In the EH group, both the PAC (*P* for trend = 0.001) and PRC (*P* for trend < 0.001) significantly declined with increasing age. Notably, the ARRs gradually increased with increasing age (*P* for trend < 0.001). In the PA group, the PRC significantly declined with increasing age (*P* for trend = 0.01), but the trends for the PAC and ARR were not significant (*P* for trend = 0.442 and 0.183, resp.) (Figure 3).

**Figure 4:**
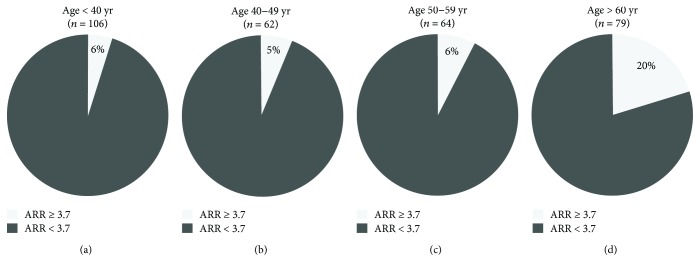
Pie charts showing the proportion of EH patients with positive or negative ARR in different age subgroups.

In the EH group, the proportion of patients with a positive ARR (ARR ≥ 3.7 (ng/dl)/(*μ*IU/ml)) was much higher in patients ≥ 60 years old than in younger patients (*P* = 0.01 versus <40 years, *P* = 0.006 versus 40–49 years, *P* = 0.014 versus 50–59 years) (Figure 4).

### 3.2. Cutoffs of the ARR for PA Screening

An ARR of 3.7 (ng/dl)/(*μ*IU/ml) showed a sensitivity of 90.7% and a specificity of 85.6% in the whole cohort. However, the accuracy of the ARR varied greatly in the different age groups (Table 3). An ARR of 3.7 (ng/dl)/(*μ*IU/ml) showed a sensitivity of 100% and a specificity of 80% in patients older than 60 years. However, with this cutoff, 10 out of 56 (18%) PA patients aged 40–49 and 5 out of 39 (13%) PA patients aged 50–59 were misdiagnosed as EH patients. Using an ARR of 3.7 as the cutoff value, the highest rate of misdiagnosis was found in PA patients younger than 40, meaning that 10 out of 39 (26%) PA patients were missed. As the ARR was used as a screening index, high sensitivity was a priority. To achieve a sensitivity higher than 90%, the optimal ARR cutoff needed to be lowered to 2.0 (ng/dl)/(*μ*IU/ml) for patients 40–49 years old and for patients 50–59 years old. With this cutoff value, the sensitivity and specificity in patients 40–49 years old were similar to those in patients 50–59 years old. The sensitivity was only 79% in patients younger than 40 years old, although the ARR was as low as 2.0 (ng/dl)/(*μ*IU/ml). To achieve a sensitivity higher than 90%, the optimal ARR cutoff value needed to be lowered to 1.0 (ng/dl)/(*μ*IU/ml) for patients younger than 40 years old. Of note, in the patients older than 60 years, ARR values of 1.0 and 2.0 resulted in low specificities of 42% and 58%, respectively.

## 4. Discussion

The present study included a relatively large number of patients to establish age-related ARR cutoffs based on the PAC and PRC, which provide valuable information for PA screening. Our study revealed that using the ARR cutoff value of 3.7 (ng/dl)/(*μ*IU/ml) was associated with a high risk of false negative results in hypertensive patients, particularly in those younger than 60 years. An ARR of 3.7 (ng/dl)/(*μ*IU/ml) is optimal for hypertensive patients older than 60 years, while for patients 40–59 years old, the optimal ARR cutoff value is 2.0 (ng/dl)/(*μ*IU/ml), and for those younger than 40 years, an ARR of 1.0 (ng/dl)/(*μ*IU/ml) may be more reasonable.

In our study, when using the ARR cutoff of 3.7 (ng/dl)/(*μ*IU/ml) for PA screening in the whole cohort, the sensitivity was 90%, which is in accordance with previous reports. In the study conducted by Burrello et al. [5], which included 75 EH patients and 20 PA patients, ARRs of 3.7, 2.7, and 1.0 (ng/dl)/(*μ*IU/ml) were reported to have sensitivities of 90%, 95%, and 100%, respectively. However, the study did not perform an age subgroup analysis due to the small sample size. Since case detection requires high sensitivity, the authors suggest an ARR between 1.0 and 2.7 (ng/dl)/(*μ*IU/ml) for PA screening. However, our data showed that a low ARR cutoff (e.g., 1–2.4 (ng/dl)/(*μ*IU/ml)) caused a high false positive rate (42%–63%) and led to unnecessary confirmatory testing, which carries the potential risk for acute volume overload in elderly patients (i.e., ≥60 years).

Several studies have focused on the effects of age on ARR screening based on the PRA but have drawn inconsistent conclusions. In a study conducted by Yin et al. [15], 216 subjects with PA and 657 subjects with non-PA were included. Similar to our results, the study found that changes in the plasma renin and ARR were more obvious in the non-PA group compared with the PA group. This phenomenon might be explained by that the effects of age on the PAC and ARR might be weakened in PA patients due to the abnormal secretion of PAC and suppression of PRC. In a study by Yin et al. [15], the subjects were divided into four age groups (≤39, 40–49, 50–59, and ≥60 years old) and the authors suggested that the criteria for the ARR needed to be set higher in the older population, but they did not recommend specific ARR cutoffs for each group. A study by Luo et al. [16] included 13 PA and 69 EH patients older than 65 years old and 32 PA and 41 EH patients younger than 65 years old. The author recommended an ARR cutoff value of 556 (PAC as pmol/l and PRA as ng/ml/h) in the elderly and an ARR cutoff value of 272 in the nonelderly. Contrary to these studies, Unger et al. [14] reported that although the number of EH patients with elevated ARR increased with advancing age, the ARR cutoffs for PA screening were not affected by age. However, when they analyzed the accuracy of the ARR in different age subgroups, only EH patients were divided according to age, while the 39 PA patients were not divided accordingly.

Our results must be interpreted in the context of the strengths and drawbacks of the study. First, although an ARR ≥ 3.7 was used as the positive cutoff in the screening protocol, many patients who tested negative also underwent the confirmatory tests, which avoided overestimating the accuracy of the ARR. Furthermore, the data were derived from a prospectively designed study and the procedures of screening and confirmation were standardized according to the guidelines. Some limitations of the study are worth mentioning. This study was carried out in a single tertiary hospital center, and the PA prevalence was higher than that in the general population in which the tests will be applied to. Nevertheless, a single-center study leads to better standardization of tests and better quality control. In addition, the study was retrospective and the PRA results were not available; therefore, we could not compare “PRC-based ARR” with “PRA-based ARR”. Based on the study protocol, 209 patients with an ARR < 3.7 who did not have any confirmatory test performed were included as EH patients. However, as our published results showed, 24 out of 135 patients (18%) with a final diagnosis of PA had an ARR < 3.7 [18]. Therefore, a small portion of the 209 patients who did not perform any confirmatory test might be misdiagnosed as EH patients, which might lead to an overestimation of the sensitivity of the 3.7 cut-off.

### 4.1. Perspectives

In summary, when an automated chemiluminescent immunoassay is used, an ARR of 3.7 (ng/dl)/(*μ*IU/ml) is associated with a high risk of false negative results in patients younger than 60 years old. An ARR of 3.7 (ng/dl)/(*μ*IU/ml) is optimal for hypertensive patients older than 60 years old; for patients 40–59 years old, the optimal ARR cutoff is 2.0 (ng/dl)/(*μ*IU/ml); and for those younger than 40 years old, an ARR of 1.0 (ng/dl)/(*μ*IU/ml) might be more reasonable.

## Figures and Tables

**Table 1:** Characteristics of patients with PA and EH.

	Essential hypertension	Primary aldosteronism	*P* value
	(*n* = 311)	(*n* = 174)	
Gender (M/F)	149/162	82/92	0.925
APA (*n*, %)	—	92, 51.7	—
Age (years)	47.77 ± 14.88	48.98 ± 12.41	0.364
Duration of HT (years)	3 (1, 7)	7 (2, 12)^∗^	0.042
History of diabetes (*n*)	72	23	0.009
History of CVD (*n*)	18	23	0.006
BMI (kg/m^2^)	25.09 (22.69, 27.7)	24.36 (22.3, 26.72)	0.024
SBP (mmHg)	152.99 ± 21.54	157.10 ± 20.64	0.042
DBP (mmHg)	93.69 ± 15.63	96.50 ± 15.38	0.047
Serum K^+^ (mmol/l)	4.0 (3.8, 4.2)	3.2 (2.7, 3.7)	<0.001
Serum Na^+^ (mmol/l)	142 (140, 143)	143 (141, 145)	<0.001
24 h Urinary Na^+^ (mmol)	140.6 (98.775, 204.75)	153.15 (112.48, 208.95)	0.244
24 h Urinary K^+^ (mmol)	33.7 (25.35, 44.65)	50.35 (34.68, 68.65)	<0.001
PAC (ng/dl)	11.00 (6.93, 17.20)	29.30 (20.20, 53.08)	<0.001
PRC (*μ*IU/ml)	17.20 (7.00, 35.40)	1.95 (0.65, 4.85)	<0.001
ARR	0.63 (0.29, 1.47)	17.19 (5.34, 54.67)	<0.001

Data were expressed as mean ± SD, %, or median (interquartile range). APA: aldosterone producing adenoma; HT: hypertension; CVD: cardiovascular disease; BMI: body mass index; SBP: systolic blood pressure; DBP: diastolic blood pressure; serum K^+^: concentration of serum potassium; serum Na^+^: concentration of serum sodium; PAC: plasma aldosterone concentration; PRC: plasma renin concentration.

**Table 2:** Characteristics of patients in different age subgroups.

	Essential hypertension (*n* = 311)				*P* for trend	Primary aldosteronism (*n* = 174)				*P* for trend
	Age < 40 y (*n* = 106)	Age 40–49 y (*n* = 62)	Age 50–59 y (*n* = 64)	Age ≥ 60 y (*n* = 79)		Age < 40 y (*n* = 39)	Age 40–49 y (*n* = 56)	Age 50–59 y (*n* = 39)	Age ≥ 60 y (*n* = 40)	
Gender (M/F)	66/40	29/33	31/33	23/56	0.000	13/26	30/26	22/17	17/23	0.133
APA (*n*, %)	—	—	—	—	—	13/26	25/31	20/19	23/17	0.165
Age (years)	31.31 ± 5.51	44.81 ± 3.18	53.86 ± 3	67.27 ± 5.78	0.000	32.15 ± 4.73	45.04 ± 2.94	54.18 ± 2.73	65.85 ± 4.22	0.000
SBP (mmHg)	155.18 ± 20.25^∗^	154.31 ± 21.41^∗^	152.06 ± 23.26^∗^	149.76 ± 21.85	0.359	158.03 ± 22.01	158.56 ± 21.13	160.23 ± 16.8	150.94 ± 21.49	0.192
DBP (mmHg)	100.7 ± 16.03	98.42 ± 11.23	92.41 ± 12.51	81.57 ± 12.83	0.000	104.9 ± 13.47	98.91 ± 14.88	95.88 ± 11.18	85.26 ± 15.24	0.000
BMI (kg/m^2^)	26.45 (23.03, 29.05)^∗^	25.54 (23.19, 27.34)	25.1 (22.96, 27.92)	24.09 (21.93, 25.91)	0.001	23.83 (21.22, 26.22)	24.51 (22.87, 27.19)	24.62 (22.83, 26.95)	24.27 (21.65, 26.45)	0.347
Serum K^+^ (mmol/l)	4 (3.8, 4.3)^∗^	4 (3.8, 4.3)^∗^	4 (3.8, 4.18)^∗^	4.1 (3.65, 4.3)^∗^	0.787	2.9 (2.7, 3.6)	3.25 (2.73, 3.8)	3.2 (2.8, 3.6)	3.1 (2.8, 3.7)	0.754
Serum Na^+^ (mmol/l)	141 (139, 143)^∗^	142 (140, 144)^∗^	142 (140, 144)^∗^	142 (140, 143)^∗^	0.211	142 (141, 145)	143 (142, 145)	143 (142, 146)	143 (141, 145)	0.374
24 h urinary Na^+^ (mmol)	130 (91.45, 182.35)^∗^	150.4 (101.45, 221.45)	148.65 (110.58, 207.25)	145.6 (96.6, 229.9)	0.262	171 (128.95, 223.8)	168.5 (110.85, 251.18)	150.6 (124.8, 188.1)	124.45 (89.05, 175.63)	0.023
24 h Urinary K^+^ (mmol)	34 (26.15, 44.9)^∗^	33 (25.43, 47.85)^∗^	28.7 (22.13, 44.14)^∗^	34.3 (27.2, 45.43)^∗^	0.422	46 (28.95, 66.45)	54.4 (35.55, 81.33)	51.4 (37.6, 64.5)	49.35 (31.25, 71.48)	0.567

Data were expressed as mean ± SD, %, or median (interquartile range). APA: aldosterone producing adenoma; BMI: body mass index; SBP: systolic blood pressure; DBP: diastolic blood pressure; serum K^+^: concentration of serum potassium; serum Na^+^: concentration of serum sodium. ^∗^*P* < 0.05 compared with corresponding PA subgroup.

**Table 3:** Accuracy of ARR for PA screening in different age subgroups.

Cutoffs	Age < 40 y (*n* = 145)		Age 40–49 y (*n* = 118)		Age 50–59 y (*n* = 103)		Age ≥ 60 y (*n* = 119)	
ARR (ng/dl)/(*μ*IU/ml)	Sen	Spe	Sen	Spe	Sen	Spe	Sen	Spe
1	0.90	0.82	0.95	0.69	0.97	0.59	1.00	0.42
1.5	0.85	0.88	0.90	0.81	0.95	0.75	1.00	0.54
2	0.79	0.91	0.90	0.84	0.95	0.80	1.00	0.58
2.4	0.77	0.92	0.86	0.89	0.89	0.80	1.00	0.63
3.7	0.74	0.94	0.82	0.95	0.87	0.94	1.00	0.80
